# Range of Motion and Muscle Activity During the Front Kick in Karate Kyokushin

**DOI:** 10.3390/jcm15041662

**Published:** 2026-02-23

**Authors:** Jacek Kaczmarski, Monika Błaszczyszyn, Zbigniew Borysiuk

**Affiliations:** Faculty of Physical Education and Physiotherapy, Opole University of Technology, 45-758 Opole, Poland; j.kaczmarski@po.edu.pl (J.K.); z.borysiuk@po.edu.pl (Z.B.)

**Keywords:** front kick, muscle activity, biomechanical analysis, EMG, neuromuscular control, kinematics

## Abstract

**Background/Objectives**: The front kick is among the most commonly used techniques in martial arts. This study aimed to analyze the range of motion during the mae-geri kick in advanced-level Kyokushin karate practitioners compared to an intermediate-level control group under three conditions: before warm-up, after warm-up, and after a shadow fight. **Methods**: The study group [N = 28, M: 27.6 years, body mass 81.9 kg, height 1.8 m] consisted of advanced-level Kyokushin karate practitioners (3rd kyu and higher), and the control group consisted of intermediate-level practitioners (6th to 4th kyu). A wireless surface electromyography (EMG) system was used to record muscle activity and an inertial measurement unit (IMU) was used to measure joint angles. Before the study began, the maximum voluntary contraction was determined for each muscle tested. Each participant performed three consecutive kicks in three conditions: before warm-up, after warm-up, and after a shadow fight. **Results**: The intermediate-level practitioners used the soleus muscle more than advanced practitioners during the front kick (48.92% vs. 35.94% before the warm-up kick, *p* = 0.042, η^2^_p_ = 0.27). After the warm-up, both groups began to use the soleus muscle more intensively (intermediate: 48.92% vs. 61.72% MVC, *p* = 0.046; advanced: 35.94% vs. 48.69% MVC, *p* = 0.045), and the advanced group’s activity in the medial gastrocnemius muscle increased compared to before the warm-up (58.23% vs. 39.20% MVC, *p* = 0.016). **Conclusions**: Advanced vs. intermediate Kyokushin karate practitioners display distinct neuro-muscular activation strategies in the mae-geri kick, particularly in soleus and gastrocnemius recruitment. Combined EMG and IMU systems can identify trends and in-form training feedback in Kyokushin karate training and effectively prepare the musculoskeletal system for rapid activity, which is important during sports competitions.

## 1. Introduction

Analyzing sports techniques according to biomechanical principles is the basis of training aimed at improving sports performance levels [1,2]. The ability to perform the correct technique in martial arts plays a key role in scoring points. Karate requires physical, technical and tactical skills and is based on striking the opponent with the hand, elbow, knee or foot. The movement patterns of these techniques usually include flexion, extension, abduction, adduction and rotation in various joints. Adequate force must be transferred to the kinetic chain in order for striking techniques to score points [3,4]. Karate athletes need to apply their force rapidly, having a high reaction time during offensive and defensive actions and being able to endure the total duration of combat (3 min) at high intensities [5].

Front kicks are particularly dangerous because they can be executed quickly and are difficult to block. One example is the mae-geri technique, which is performed above the waist in the abdominal area or celiac plexus. It is one of the most common techniques for direct elimination or maintaining the right distance [6,7]. Mae-geri is a ballistic movement in which the attacker attempts to strike the opponent with their foot. Proper neuromuscular conduction, anticipation, and proprioception are required to perform this movement correctly. For advanced karate practitioners, the mae-geri pattern involves hip flexion with adduction and internal rotation, followed by knee extension and plantar flexion of the foot with slight external rotation. Learning the correct movement pattern enables appropriate training, especially in the initial phase, as well as injury prevention and better results during competitions [8,9,10,11,12,13].

The power and speed of a kick depend mainly on the number of muscles or muscle fibers involved in the work [14,15]. Analyzing data from selected articles, it was found that the force of a kick may be related to the isokinetic strength of the hip flexors and extensors and the angular velocity of the knee joint [16,17]. An additional factor determining success is the reaction time in response to a stimulus, which differs significantly between be-ginners and advanced karate practitioners [18,19,20,21,22,23,24,25].

Previous studies have analyzed the kinematics and muscle activation of the mae-geri kick, including comparisons across skill levels [2,4,6,10]. However, limited attention has been paid to how different preparatory conditions (warm-up vs. fatigue-like states) modulate neuromuscular strategies across expertise levels using synchronized sEMG and IMU measurements.

Physiologically, warming up induces a number of changes, such as an increase in muscle temperature, increased peripheral blood flow, and subsequent increased muscle fiber performance, primarily due to the phenomenon of post-activation performance potentiation (PAPE) [5,26]. A typical warm-up includes exercises to prepare the musculoskeletal system for intense and dynamic activities (running and static stretching), as well as specific exercises by adding dynamic stretching and high-intensity exercises specific to Kyokushin karate. The impact of warm-ups on the physical fitness and combat ability of kumite athletes remains largely unclear. Over the past few decades, warm-ups with external loads (e.g., vests, resistance bands) have been a popular training strategy in many sports [5].

Coaches can use various warm-up strategies before competitions that can significantly improve kumite performance. One method used in karate is so-called “shadow fight” or “shadowboxing”. In the martial arts, “shadowboxing” has been defined by Croom [27] as: “the practice of rehearsing and refining martial arts techniques and mentally simulating training or combat-relevant scenarios in order to develop technical mastery and physical capacity”.

Shadow fight exercises effectively strengthen motor memory, improve neuromuscular coordination and combat readiness without the risk of injury. Shadow fight exercises are used by athletes of all fighting styles, including boxing, capoeira, karate, kickboxing, krav maga, kung fu, sambo and taekwondo, mainly as individual practice of technical forms, involves imagination, mental simulation, planning, and complex and accurate motor execution [28,29,30].

The main objective of this study was to analyze the mae-geri kick movement pattern, range of motion and skeletal muscle activation during the execution of a mae-geri front kick performed by intermediate and advanced karate practitioners under three conditions: before warm-up, after warm-up, and after a shadow fight. The combination of electromyography (EMG) systems and inertial sensors enabled an objective biomechanical analysis of the mae-geri kick, considering the working muscles and the temporal and spatial parameters of the movement. Analyzing the movement pattern of the mae-geri kick is essential for injury prevention, technique application, and research.

Main hypotheses: 1) Athletic advancement determines differences in movement patterns in the mae-geri kick under three conditions: before warm-up, after warm-up, and after a shadow fight. 2) Biokinetic parameters of the mae-geri pattern in athletes at different levels of advancement change in three measurement conditions, i.e., before warm-up, after warm-up, and after shadow boxing, with greater differences expected in athletes at an intermediate level of training compared to athletes at an advanced level.

## 2. Materials and Methods

### 2.1. Participants

The study was conducted from September to December 2023 in the Human Movement and Biomechanics Laboratory at the Faculty of Physical Education and Physiotherapy at Opole University of Technology. The study group consisted of 13 (age 31 ± 11) advanced-level Kyokushin karate practitioners (3rd kyu and higher) and a control group of 15 (age 24 ± 7) intermediate-level karate practitioners (from 6th to 4th kyu) (Table 1). All participants were Caucasian males and from southern Poland. Each participant underwent anthropometric measurements. The Seca 515 mBCA Hamburg

Deutschland device with Seca analytics 115 software and the Seca 285 height meter were used to determine BMI (body mass index). Prior to the study, each participant was informed of the study’s purpose and assumptions and provided written consent to participate. The study design included inclusion and exclusion criteria based on two main elements: the athletes’ performance level and their overall good health, with no factors limiting the execution of the analyzed technique. Participants with current injuries, musculoskeletal disorders, or medical conditions that could affect movement performance were excluded. Group classification into advanced and intermediate karate practitioners was based on performance level and coach verification. Each testing session was preceded by an interview with the athlete to confirm eligibility. Inclusion criteria for both groups: age 18 years or older, no injury in the last 6 months, 6th kyu or higher level of Kyokushin karate, active participation in training at least twice a week, no health infections in the last 3 months, no significant weight fluctuations in the last 3 months. Exclusion criteria: age under 18 years, karate level below 6th kyu, injury in the last 6 months, significant weight loss or gain in the last 3 months, training breaks in the last 3 months, and a current health infection in the last 3 months.

**Figure 1:**
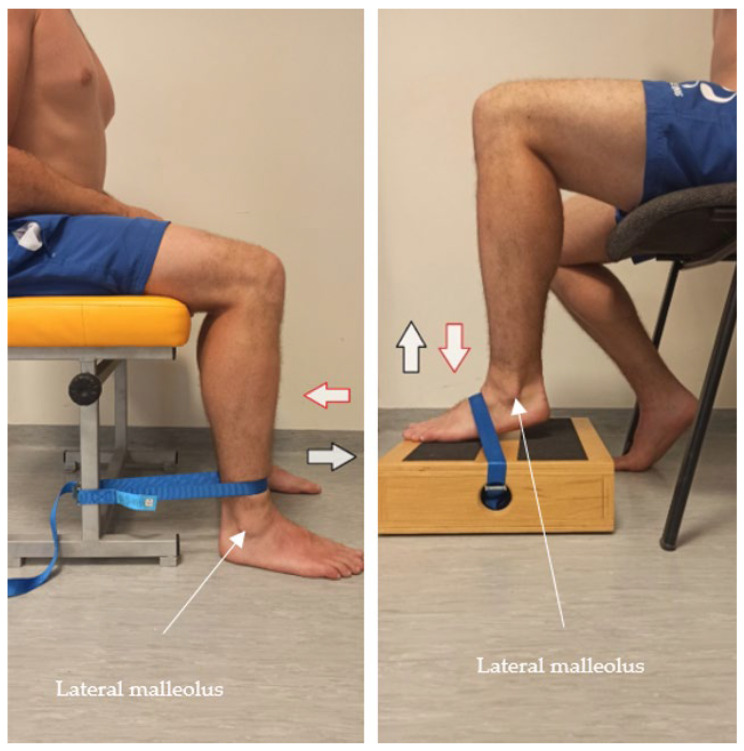
MVC position for the rectus femoris, vastus lateralis, and soleus muscles. Red arrow—resistance; black arrow—movement against resistance.

**Figure 2:**
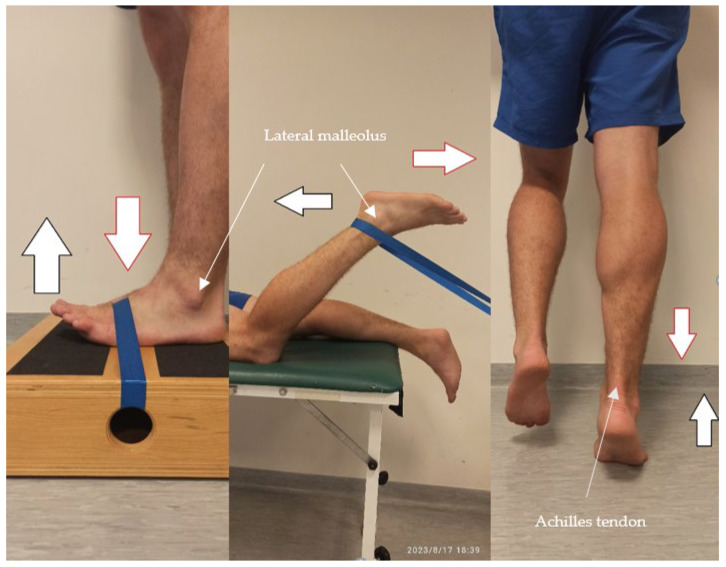
MVC position for the tibialis anterior and biceps femoris, the medial gastrocnemius and the lateral gastrocnemius. Red arrow—resistance; black arrow—movement against resistance.

Before the tests began, the maximum voluntary contraction (MVC) was determined for each muscle. Participants performed a movement against resistance, holding the position for three seconds. This was followed by a pause, then another three seconds of tension. The following muscles were selected for testing: the rectus femoris, vastus lateralis, and soleus (Figure 1), the tibialis anterior, the biceps femoris, the medial gastrocnemius and the lateral gastrocnemius (Figure 2) (the absence of the gluteus maximus and medius muscles was due to technical problems. MVC positions were chosen based on muscle function: gastrocnemius (biarticular) with the knee extended, soleus (uniarticular) with the knee flexed (90° to minimize gastrocnemius involvement. The large number of kicks, the energetic nature of mae-geri, and the long duration of the study caused problems with the proper placement of the electrodes).

The MVC testing positions for the lower limbs are shown below.

Each karate practitioner performed three consecutive kicks under three conditions: before warming up, after warming up, and after a shadow fight (90 s). For each condition, participants kicked three times in the air (A) and three times at a kick pad (P). The target area was the chūdan, or the space above the waist and below the shoulders. Participants kicked a pad held by an examiner, who signaled the start of each kick by moving the pad. This allowed the reaction time to be determined. The participants performed three consecutive kicks, without any clear breaks, but with the starting and finishing positions marked. The kick against the pad began with a reaction to the movement of the pad held by the coach, with breaks of no more than 10 s. The procedure included the following: (1) three mae-geri kicks in the air, (2) three mae-geri kicks to the shield, (3) warm-up—10 push-ups, 10 squats, 15 push-ups, 15 squats, (4) three mae-geri kicks in the air, (5) three kicks to the shield, (6) “Shadow fighting”—block, punch, kick for 1.5 min, 30 s break, and 1.5 min of shadow fighting, (7) three mae-geri kicks in the air, and (8) three mae-geri kicks to the shield. Immediately after the warm-up, the participant assumed a kicking position, similarly after shadow fight. The results of the three kicks were averaged to give each participant one score for each condition: before warm-up (A1 and P1), after warm-up (A2 and P2), and after a shadow fight (A3 and P3). Any data with errors or artifacts were excluded from the analysis.

Immediately after the warm-up, the participant assumed a kicking position, similarly after shadow fight. The results of the three kicks were averaged to give each participant one score for each condition: before warm-up (A1 and P1), after warm-up (A2 and P2), and after a shadow fight (A3 and P3). Any data with errors or artifacts were excluded from the analysis.

### 2.2. Measurements

#### 2.2.1. Surface Electromyography (sEMG) 

The study used a wireless sEMG direct transmission system (Noraxon, Scottsdale, AZ, USA) with a sampling rate of 3000 Hz to record muscle bioelectrical activity. The digital EMG signal was transmitted telemetrically to a PC. Signal processing and analysis were performed using MyoResearch 3.16 software. The signal was filtered using a bandpass filter with cutoff frequencies of 80–250 Hz, and then smoothed using root mean square (RMS) with a time window of 100 ms. Pre-gelled, self-adhesive surface electrodes (Ag/AgCl, Sorimex, Toruń, Poland) were placed along the longitudinal midline of the muscle, between the point of movement and the tendon attachments, in accordance with SENIAM guidelines. Before placing the electrodes, the participant’s skin was prepared. Hair was removed and the skin was disinfected and dried. The body sensors were attached with double-sided adhesive tape, and the entire assembly, including the electrodes, was secured with the Noraxon straps included in the kit. A total of seven sensors were placed on each tested muscle. The EMG signal was normalized to MVC and then averaged.

#### 2.2.2. Inertial Measurement Unit (IMU)

A wireless inertial sensor system (Mymotion, Noraxon), consisting of five IMU sensors with a sampling rate of 200 Hz, was used to measure the joint angles and movement of the hand holding the kick pad. Signal analysis was performed using myoResearch 3.16 software. Four sensors were attached to the participant’s body with the Noraxon straps and one was placed on the examiner’s wrist holding the kick pad. An accelerometer on the examiner’s wrist measured the reaction time when kicking a target. The participant kicked at the moment the pad moved forward. Reaction time was determined from the first signal from the wrist accelerometer to the participant’s first foot movement. Movement time was determined from the beginning of foot movement to maximum knee joint extension, at a clear signal change. Four inertial sensors, located on the pelvis, thigh, lower leg, and foot, were used to determine angular values. The angles of the initial and final positions of the hip (flexion–extension, abduction–adduction, and internal–external rotation), knee (flexion–extension, abduction–adduction, and internal–external rotation), and foot (plantar flexion–dorsiflexion, abduction–adduction, and inversion–eversion) were measured during the kick.

### 2.3. Statistical Analysis

ANOVA (analysis of variance) was used for statistical analysis, with repeated measures of start-to-end of movement (S-E), conditions (A1, A2, A3, P1, P2, P3), and group (advanced or intermediate). An inter-group comparison of muscle activity in the three conditions was performed using Tukey’s post hoc test. Normality of distribution was checked with a Q-Q plot; equality of variances was tested with Levene’s test; and sphericity with Mauchly’s test. The Greenhouse-Geisser correction was used to adjust for the lack of sphericity. The level of statistical significance was set at α = 0.05. The analysis was performed using Jamovi 2.3.24.0 software.

## 3. Results

Statistically significant angular values and percent maximum voluntary contraction (%MVC) were found within groups of karate practitioners performing a front air kick.

**Figure 3:**
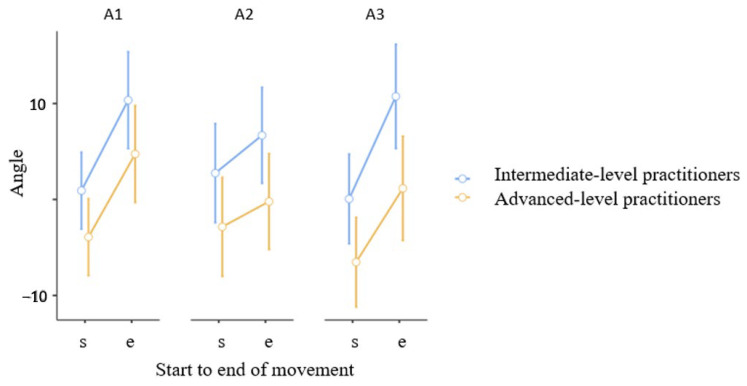
Angular values (s—start to e—end) of knee rotation in three conditions (A1, A2, A3) during an air kick. Mean, CI.

Figure 3 shows the angular values of knee joint rotation during an air kick. The advanced-level group demonstrated statistical significance, with the knee joint being externally rotated during the final phase of the kick before warm-up A1 (4.74° ± 11.99°), and internally rotated after the warm-up A2 (−0.21° ± 11.28°). Significant main effects were found between S-E (F = 39.50, *p* < 0.001, η^2^_p_ = 0.62), S-E x Time (F = 5.53, *p* = 0.007, η^2^_p_ = 0.18) and Group (F = 5.92, *p* = 0.001, η^2^_p_ = 0.19, indicating a large effect size) Thus, the knee joint’s movement pattern changed. The statistical significance was *p* = 0.026. No other results reached statistical significance.

**Figure 4:**
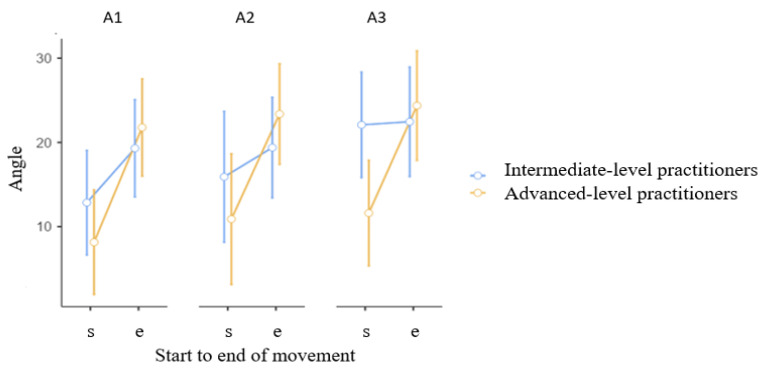
Angular values of knee flexion (s—start to e—end) in three conditions (A1, A2, A3) during an air kick. Mean, CI.

Figure 4 illustrates the range of motion of the knee joint in three conditions (A1, A2, A3) during a kick for advanced- and intermediate-level karate practitioners. Significant differences in knee flexion were observed in the starting position in the intermediate group. Before warming up (A1), the knee joint flexed at an angle of 12.84° ± 9.96°. After shadow fighting (A3), however, significantly greater flexion was observed in the starting position (22.08° ± 11.57°). There was a significant interaction between S-E (F = 15.94, *p* < 0.001, η^2^_p_ = 0.39), S-E × Group (F = 5.381, *p* = 0.029, η^2^_p_ = 0.18) and Time (F = 4.87, *p* = 0.014, η^2^_p_ = 0.16, indicating a large effect size). This difference was statistically significant at *p* = 0.041. No other results reached statistical significance.

**Figure 5:**
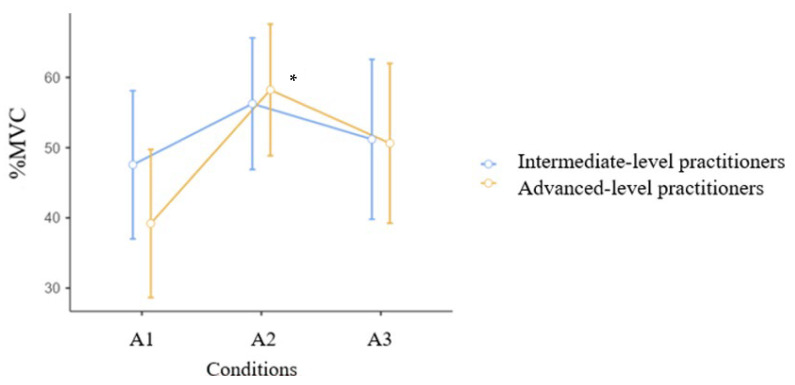
MVC (%) for the medial gastrocnemius muscle in three conditions (A1, A2, A3) during an air kick. Intermediate n = 15, Advanced n = 13. Mean, CI. * *p* = 0.016.

Figure 5 shows the activation of the medial gastrocnemius muscle during an air kick in three conditions. Significant main effects were found for Time (F = 6.77, *p* = 0.003, η^2^_p_ = 0.22, indicating a large effect size). The time × group interaction was not statistically significant (F= 1.02, *p* = 0.368, η^2^_p_ = 0.041), and for group was also not statistically significant (F = 0.17, *p* = 0.687, η^2^_p_ = 0.007).” After warm-up, it was observed that the advanced group (A2) began to engage the muscle significantly more 58.23 ± 15.10 vs. 39.20 ± 19.07%MVC, at *p* = 0.016 *.

**Figure 6:**
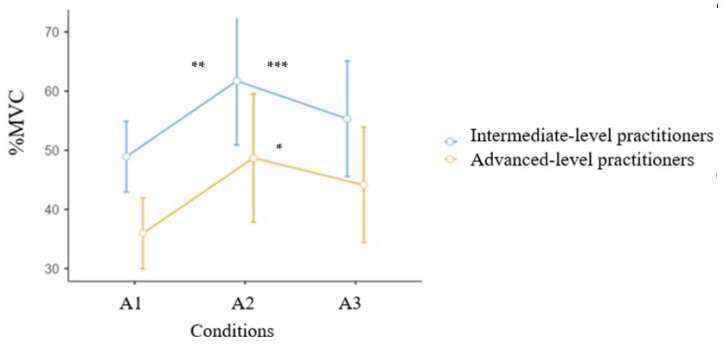
MVC (%) for the soleus muscle in three conditions during a front kick. Intermediate n = 15, Advanced n = 13. Mean, CI. * *p* = 0.046, ** *p* = 0.045, *** *p* = 0.042.

Figure 6 shows the activation of the soleus muscle in both groups during an air kick in three conditions. Significant main effects were found for Group (F = 9.2, *p* < 0.001, η^2^_p_ = 0.27, indicating a large effect size). There was also a significant interaction between Group and Time (F = 5.69, *p* = 0.02, η^2^_p_ = 0.19, F = 9.20, *p* < 0.001, η^2^_p_ = 0.277, indicating a large effect size). The time × group interaction was not statistically significant (F = 0.06, *p* = 0.941, η^2^_p_ = 0.003). After the warm-up (A2), both groups used the soleus muscle significantly more than before. A statistically significant difference was found in the advanced group between the conditions (35.94 ± 12.37 vs. 48.69 ± 15.09%MVC, *p* = 0.046 *), while in the intermediate group, the difference was 48.92 ± 8.07 vs. 61.72 ± 22.13%MVC, at *p* = 0.045 **. Additionally, intermediate-level practitioners used the soleus muscle significantly more (A1) than advanced-level practitioners, which was statistically significant at *p* = 0.042 ***.

**Figure 7:**
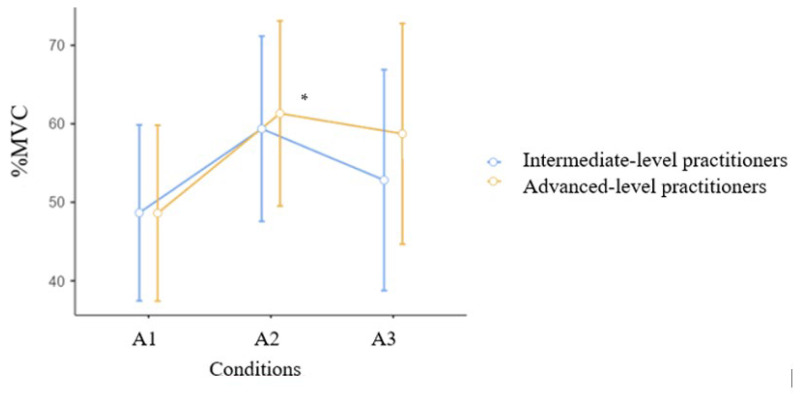
MVC % for the lateral gastrocnemius muscle during the front kick in three conditions. Intermediate n = 15, Advanced n = 13. Mean, CI. * *p* = 0.024.

Figure 7 shows the activation of the lateral gastrocnemius muscle in both groups during an air kick in three conditions. The intermediate group used the muscle at 48.66 ± 13.89%MVC in condition A1 and 59.3 ± 16.9%MVC in condition A2. The advanced group had similar results (48.61± 23.96 and 61.32 ± 23.72%MVC, respectively). Significant main effects were found for Time (F = 7.59, *p* = 0.001, η^2^_p_ = 0.24, indicating a large effect size). Neither the time × group interaction (F = 0.50, *p* = 0.609, η^2^_p_ = 0.020) nor the main effect of group (F = 0.11, *p* = 0.740, η^2^_p_ = 0.005) was statistically significant. Advanced athletes, after warm-up, shift toward gastrocnemius activation, suggesting efficiency gains. This latter result was statistically significant at *p* = 0.024 *.

## 4. Discussion

The science and practice of combat sports are undergoing a profound transformation, driven by the convergence of biomechanics and wearable sensor technology [31]. Rapid, multidirectional movements, including high accelerations, pose a particular challenge for inertial-based motion reconstruction, with limited task-specific validation studies available [32,33,34]. Understanding biomechanics parameters is fundamental, as their quantification allows for the objective evaluation of technique, the optimization of performance, and the identification of movement patterns that may increase the risk of both acute and chronic injury [31]. Complementing kinetic and kinematic analyses, sEMG provides a window into the neuromuscular control underpinning motor control.

Warm-up exercises are commonly used before training or competitions, and their influence on the activity of athletes seems obvious; however, there are few scientific studies on the influence of these exercises on maintaining the movement pattern in karate. The study showed significant differences in the activation of the medial gastrocnemius muscle during an air kick in three conditions (Figure 5). After warm-up, it was observed that the advanced group began to engage the muscle significantly more than the intermediate group. The exercise program that included push-ups, squats, and karate techniques performed as shadow fighting resulted in increased muscle activation. In the analysis of the soleus muscle (Figure 6), a statistically significant difference was found in both group between the conditions (A1, A2, A3). Figure 7 shows the activation of the lateral gastrocnemius muscle in both groups during an air kick in three conditions. Similar to the soleus muscle, both groups activated the lateral gastrocnemius muscle more after the warm-up (Table 2). As reported by [35] during warm-up, muscle temperature increases, accompanied by increased muscle metabolism and muscle fiber conduction. An acceleration of VO2 kinetics and increased muscle contraction efficiency after previous contraction were also found.

In this study, an increase in muscle activation normalized to maximum voluntary contraction (MVC) was obtained for the biceps femoris, soleus, and gastrocnemius muscles. There was a higher percentage of MVC activation after the squat/push-up cycle and a slightly lower percentage after the shadow boxing cycle (Table 2). Reference [36] also reported increased muscle activity in the vastus lateralis and rectus femoris muscles. These results suggest that warm-up exercises, such as flexible resistance kicks, positively impact kicking performance in karate. Biomechanics indicators as attributes of kick effectiveness against a target. In karate training, explosive strength is essential for delivering powerful strikes and performing fast movements. To improve strength and speed, it is worth incorporating exercises that engage key muscle groups into training. These exercises generate greater strength at maximum speed, enabling fighters to execute lightning-fast punches, kicks, and evasive techniques [37]. In the present study, advanced karate practitioners straightened their knee joints and flexed their feet plantarly during kicks against a pad. This reflects changes in gastrocnemius and soleus muscle activation. The advanced-level group demonstrated statistical significance (Table 2)the knee joint being externally rotated during the final phase of the kick before warm-up, and internally rotated after the warm-up (Figure 3). Figure 4 illustrates the range of motion of the knee joint in three conditions (A1, A2, A3) during a kick for advanced- and intermediate-level karate practitioners. Significant differences in knee flexion were observed in the starting position in the intermediate group before warming up (A1). After shadow fighting, however, significantly greater flexion was observed in the starting position. Data from selected articles revealed that the impact force of a karate kick may be related to the isokinetic strength of the hip flexors and extensors, as well as the angular velocity of the knee joint [38,39]. Therefore, it is advisable to improve kicking strength through training that includes functional exercises focused on explosive lower-limb strength. In addition to traditional exercises, these exercises should emphasize movements that support the pre-contact and contact phases of the kick [40,41]. Comparisons between experienced karateka and novices reveal significant differences in neuromuscular strategy; experts exhibit more refined activation timings and shorter muscle contraction periods, which are indicative of greater neuromuscular efficiency and coordination honed through years of practice [31]. To achieve the maximum foot velocity in executing a front kick, athletes must increase the velocity of the knee as it travels toward the target. On the other hand, for the optimal execution of a straight kick, the axial over-rotation of the hips in the sagittal plane becomes necessary to generate a higher impact force. Regarding the roundhouse kick, the velocity of the kicking foot results from the combined effect of linear motion at the pivot hip and the angular motion of the pelvis around the pivot hip, with the hip displacement contributing significantly in the initial phases [25,40]. sEMG assesses muscle activity, and inertial sensors describe the position of body segments in space. Together, they can precisely determine differences and similarities in movement patterns. A comprehensive assessment at the beginning of a martial arts practitioner’s career may support skill acquisition. Correcting practitioners as they acquire new skills may support skill acquisition and these tools may help identify risk factors or guide training adjustments. When kicking in the air, the lower limb generates movement through the hip, knee, and ankle joints, “driving” the subsequent segments: the thigh, lower leg, and foot. Ideally, the final element of the biomechanical chain, the foot, will reach the target with maximum force and speed, “resting” on the opponent—in this case, the chudan. However, by kicking in the air, this “rest” is not achieved. Magalhaes et al. [38] observed that a standard warm-up program significantly improved the angular position of the knee joint in a closed kinematic chain when applied before activity in karate practitioners. The complex motor information, provides awareness of the body’s position in space. Clearly, any functional changes in muscles, which control joints, play a key role in perception of position in open or closed kinematic chains. Deep sensation plays a key role in conscious and unconscious sensations, automatic movement control, coordination, balance, and motor learning [38]. Thus, improving proprioception through warm-up exercises can influence the correct positioning of body segments in space, reducing the risk of injury [39]. In this study, an increase in muscle activation normalized to MVC was observed for the following muscles: biceps femoris, soleus, and gastrocnemius. However, %MVC activation was higher after the squat/push-up cycle and slightly lower after “shadow boxing.” Aandahl et al. also noted an increase in muscle activity in the vastus medialis and rectus femoris. These results indicate the positive impact of warm-up training, including kicking with elastic resistance, on karate kicking performance [36]. This finding supports the use of active rest strategies (involving kata techniques) to maintain and improve motor readiness especially in young karate athletes [41].

### Practical Recommendations

Analyzing data from selected articles, it was found that kicking technics may be related to the isokinetic strength of the hip flexors and extensors and the angular of the knee joint. Therefore, it is worth recommending improving kicking power through training that includes functional exercises focused on explosive strength of the lower limbs, which, in addition to traditional exercises, also focus on exercises that support the pre-contact and contact phases of the kick.

## 5. Conclusions

Advanced vs. intermediate Kyokushin karate practitioners display distinct neuro-muscular activation strategies in the mae-geri kick, particularly in soleus and gastrocnemius recruitment. Combined EMG and IMU systems can identify trends and in-form training feedback in Kyokushin karate training and effectively prepare the musculoskeletal system for rapid activity, which is important during sports competitions.

## 6. Limitations

The inclusion criteria for the study included selecting athletes from two different skill levels and the same karate style, which significantly limited the number of participants. An additional limitation was the adopted research protocol and the research equipment used, which required multiple repetitions and the organization of the study time and location. Therefore, the study included athletes training in specific karate clubs. In the future, it is worthwhile to examine the trajectory of upper limb kicks and punches, as well as muscle bioelectric activity, across different age groups, extreme skill levels, and by gender. The absence of EMG data for the gluteus maximus and medius was due to technical limitations. The high number of kicks, the dynamic nature of the mae-geri technique, and the long duration of the sessions caused the electrodes to detach in these areas. Additionally, the anatomical location of these muscles required that sensors and electrodes be secured with special tapes, which restricted movement. For these reasons, it was decided to omit the hip stabilizers from the study. One of the limitations of the present study is the variation in age between the groups. While this does not compromise the primary focus of the research, which was the level of athlete training, it is acknowledged as a characteristic that should be considered in the interpretation of the findings.

## Figures and Tables

**Table 1:** Characteristics of intermediate and advanced karate practitioners.

Karate Practitioner	Age [Years]	Body Mass [kg]	Body Height [m]	BMI	Rank	Leg Dominance
1i	21	87	1.78	27.46	6th kyu	R
2i	37	93	1.79	29.03	6th kyu	R
3i	21	63	1.72	21.30	6th kyu	R
4i	35	84	1.82	25.36	5th kyu	R
5i	18	70	1.70	24.22	6th kyu	R
6i	19	83	1.85	24.25	6th kyu	R
7i	20	67	1.82	20.23	5th kyu	R
8i	18	65	1.75	21.22	6th kyu	R
9i	23	74.5	1.82	22.49	5th kyu	R
10i	20	93	1.96	24.21	6th kyu	R
11i	22	78	1.83	23.29	5th kyu	R
12i	21	114	1.80	35.19	4th kyu	R
13i	39	90	1.84	26.58	6th kyu	R
14i	23	73	1.80	22.53	4th kyu	R
15i	30	78	1.79	24.34	5th kyu	R
1a	22	85	1.80	26.23	3rd kyu	R
2a	37	84	1.82	25.36	1st dan	R
3a	41	96	1.82	28.98	2nd kyu	R
4a	34	105	1.83	31.35	1st dan	R
5a	22	66.5	1.79	20.75	2nd kyu	R
6a	55	89	1.82	26.87	3rd dan	R
7a	43	89.5	1.85	26.15	2nd kyu	R
8a	20	64	1.70	22.15	3rd kyu	R
9a	18	75	1.80	23.15	1st kyu	R
10a	24	91.5	1.86	26.45	1st dan	R
11a	24	74	1.75	24.16	2nd kyu	R
12a	38	83	1.82	25.06	2nd kyu	R
13a	28	73	1.78	23.04	2nd kyu	R

i—intermediate, a—advanced, R—right.

**Table 2:** Statistically significant results obtained during the mae-geri kick in karate practitioners (Tukey’s post hoc test).

Variable/Measure	A1 Mean ± SD	A2 Mean ± SD	A3 Mean ± SD	Mean Diff	SE	df	t	*p* (Tukey)
Knee joint rotation—advanced (°)	4.74 ± 11.99	−0.21 ± 11.28	–	4.95	1.27	24	3.90	0.026
Knee flexion—intermediate (°)	12.84 ± 9.96	–	22.08 ± 11.57	−9.24	2.50	24	−3.69	0.041
Medial gastrocnemius—advanced (%MVC)	39.20 ± 19.07	58.23 ± 15.10	–	−19.03	5.28	24	−3.60	0.016
Soleus—advanced (%MVC)	35.94 ± 12.37	48.69 ± 15.09	–	−12.75	4.08	24	−3.13	0.046
Soleus—intermediate (%MVC)	48.92 ± 8.07	61.72 ± 22.13	–	−12.80	4.08	24	−3.14	0.045
Soleus—advanced vs. intermediate (%MVC)	35.94 ± 12.37 vs. 48.92 ± 8.07	–	–	12.98	4.10	24	3.17	0.042
Lateral gastrocnemius—advanced (%MVC)	48.61 ± 23.96	61.32 ± 23.72	–	−12.71	3.72	24	−3.42	0.024

SD—standard deviation, *p*—statistical significance, MVC—maximal voluntary contraction.

## Data Availability

The original contributions presented in this study are included in the article. Further inquiries can be directed to the corresponding author.
